# Clinical value evaluation of MEG3/miR-144-3p as a biomarker for the early diagnosis of glaucoma

**DOI:** 10.3389/fimmu.2026.1894482

**Published:** 2026-10-20

**Authors:** Tony Yihao Chen, Shuwei Chen, Na Wu, Xinghuai Sun

**Affiliations:** 1 Department of Ophthalmology and Visual Science, Eye and ENT Hospital, Shanghai Medical College, Fudan University, Shanghai, China; 2 NHC Key Laboratory of Myopia, Fudan University, Shanghai, China; 3 Eye Institute, Eye and ENT Hospital, College of Medicine, Fudan University, Shanghai, China; 4 Department of Plastic Surgery, Zhongshan Hospital, Fudan University, Shanghai, China; 5 Key Laboratory of Myopia, Chinese Academy of Medical Sciences, National Health Commission, Shanghai, China; 6 Shanghai Key Laboratory of Visual Impairment and Restoration, Science and Technology Commission of Shanghai Municipality, Shanghai, China; 7 State Key Laboratory of Medical Neurobiology, Institutes of Brain Science and Collaborative Innovation, Center for Brain Science, Fudan University, Shanghai, China

**Keywords:** early POAG, glaucoma, inflammatory factors, MEG3, miR-144-3p

## Abstract

**Purpose:**

LncRNA MEG3 and miR-144-3p have been confirmed to be related to ocular pathological processes, but their roles as early diagnostic biomarkers for Primary open angle glaucoma (POAG) and the underlying regulatory mechanisms remain unclear.

**Methods:**

The targeting relationship between MEG3 and miR-144-3p was verified by the dual luciferase reporter gene assay. MEG3 and miR-144-3p expression were detected by real-time quantitative polymerase chain reaction (RT-qPCR). ROC curves and correlation analysis were used to evaluate the diagnostic value and correlation of MEG3 and miR-144-3p. NaIO_3_ was used to treat retinal ganglion cells (RGCs), CCK-8 was used to measure cell proliferation and survival rate, and ELISA was used to detect levels of CAT, MDA, and inflammatory factors (IL-6, TNF-α, IL-1β).

**Results:**

MEG3 and miR-144-3p have a targeting relationship. Compared with the healthy control group, the expression of MEG3 in tears and serum of patients with early POAG was significantly increased, while the expression of miR-144-3p was decreased. ROC analysis showed that MEG3 (serum AUC = 0.899; tears AUC = 0.894) and miR-144-3p (serum AUC = 0.877; tears AUC = 0.873) had excellent diagnostic efficacy for early POAG. In RGCs, silencing MEG3 or overexpressing miR-144-3p could alleviate NaIO_3_-induced cell damage, inflammation, and oxidative stress.

**Conclusion:**

MEG3 and miR-144-3p form a regulatory axis involved in glaucoma pathogenesis. Their abnormal expression in tears and serum makes them promising non-invasive biomarkers for early POAG diagnosis. The aqueous humor samples used in the study were solely for the analysis of the correlation of regulatory mechanisms.

## Introduction

Glaucoma is a major blinding eye disease that causes permanent vision loss worldwide (1). According to statistics from World Health Organization, glaucoma has affected over 80 million people (2). Primary open angle glaucoma (POAG) is the most common type of glaucoma, accounting for 60-70% of all cases of glaucoma (3). The pathological process of POAG is regulated by multiple factors, including increased intraocular pressure (IOP), optic nerve degeneration, intensified oxidative stress, and damage of retinal ganglion cells (RGCs) (4, 5). However, the deep molecular regulatory network has not yet been fully elucidated (6). What’s more serious is that early POAG usually lacks specific clinical manifestations, resulting in delayed diagnosis (7, 8). At the same time, existing non-invasive detection techniques suffer from insufficient sensitivity, which causes most patients to be diagnosed at an advanced stage and miss the optimal window for intervention (9). Therefore, the development of new molecular biomarkers is crucial for the early detection and treatment of POAG.

Long non-coding RNAs (lncRNAs) are non-coding RNA molecules whose nucleotide length exceeds 200 nucleotides (10, 11). Their core functions in the gene regulation network and the pathological process of diseases have become a current research hotspot (12). In recent years, more and more research evidence has shown that the imbalance of lncRNA expression is closely related to the occurrence and development of glaucoma (13, 14). Existing studies have confirmed that various lncRNAs participate in the pathological physiological processes related to POAG by regulating different signaling pathways (15). For example, the down-regulation of lncRNA GAS5 can promote the survival and differentiation of RGCs by activating the TGF-β signaling axis (16). LncRNA MALAT1 exerts an anti-apoptotic effect through the PI3K/Akt pathway and protects RGCs in the glaucoma state (17). It is worth noting that lncRNA MEG3 has also been confirmed to have ocular pathological regulatory functions. It can regulate neuroinflammation and mediate neuroprotective mechanisms in retinal ischemia-reperfusion injury (18). In addition, MEG3 shows significantly high expression in clinical specimens of diabetic retinopathy patients and in human retinal microvascular endothelial cell models induced by high glucose (19). However, the specific role and regulatory network of this molecule in the course of glaucoma still need to be further analyzed.

In addition to lncRNA, microRNA (miRNA) is also an indispensable key regulatory molecule in the pathogenesis of glaucoma (20). These two often form a synergistic regulatory pattern by constructing competitive endogenous RNA (ceRNA) networks, and jointly participate in the physiological and pathological processes of the eye (21). Multiple studies have found that in the serum samples and retinal tissues of glaucoma patients, various miRNAs such as miR-128, miR-445-3p, and miR-21-5p exhibit abnormal expression (22, 23). These miRNAs are believed to participate in the disease progression of glaucoma by regulating key pathological processes such as inflammatory responses and maintaining IOP homeostasis (24, 25). Among them, the CCK-8 experiment results show that overexpression of miR-144 can significantly enhance the activity of RGCs and effectively alleviate the decline in cell viability induced by oxidative stress (26). As a member of the miR-144 family, miR-144-3p shows a significantly downregulated expression in the lesion tissues of glaucoma patients and in human scleral fibroblasts treated with transforming growth factor-β1 (TGF-β1) (27). We discovered that MEG3 and miR-144-3p have interaction targets through bioinformatics analysis. This provides important clues for studying the collaborative regulatory mechanism of these two factors in POAG.

This study aims to investigate the expression of MEG3 and miR-144-3p in the AH, tears and serum samples of early POAG patients. It also seeks to explore the molecular regulatory mechanisms of MEG3 and miR-144-3p in the pathogenesis of early POAG, providing theoretical basis and experimental evidence for the development of new diagnostic markers for POAG and the formulation of targeted treatment strategies.

## Materials and methods

### Subjects

A retrospective analysis was conducted on 63 patients with early POAG (n=63, MD > -6 dB) and 55 patients with mid-late POAG (n=55, MD ≤ -6 dB). At the same time, 52 healthy individuals with matching age and gender were selected as the healthy control group (HC). The subjects in the HC group were all excluded from having POAG or any other organic eye disorders. This study was approved by the ethics committee of Eye and ENT Hospital, Shanghai Medical College, Fudan University, and strictly followed the principles of the Declaration of Helsinki and relevant medical ethics norms. All participants signed informed consent forms.

### Baseline information collection

The collection of baseline data and specific ocular indicators generally includes age, gender, IOP, mean defect of visual field (MD), and retinal nerve fiber layer thickness (RNFL). All ocular examinations were performed by the same experienced ophthalmologist. The MD examination was performed using an automatic perimeter (HFA; Carl Zeiss-Meditec, USA), RNFL detection was carried out using an optical coherence tomography (OCT) instrument, and IOP measurement was done with a non-contact tonometer. This ensured the accuracy, consistency, and comparability of all examination results.

### Sample collection

All serum and tear samples were collected from the subjects before they received any glaucoma-related treatment or surgical intervention. The collection process strictly followed aseptic operation protocols and was carried out by professional medical staff. Serum samples were collected from the elbow veins of all participants, with a volume of 5 mL. These samples were then stored in a -80°C refrigerator for cryopreservation to prevent repeated freezing and thawing. The tear samples were collected using a sterile capillary tube. The sterile capillary tube was gently placed in the conjunctival sac of the lower eyelid of the subject to naturally aspirate the tear. The collection volume was approximately 50-100 μL. After collection, the sample was immediately transferred to a sterile centrifuge tube, centrifuged at low temperature, and the supernatant was aliquoted and frozen at -80 °C for future use. The AH samples were only collected from the patients in the POAG group. The collection was performed during the POAG-related surgery (such as trabeculectomy), using a sterile syringe to slowly and gently extract 50-80 μL of AH. After collection, the sample was quickly injected into a sterile cryotube and protected from light. It was then frozen at -80 °C in an ultra-low temperature refrigerator.

### RT-qPCR

The RNA extraction was carried out using the miRNeasy Serum/Plasma Advanced kit (Qiagen, Germany). RNA purity was assessed using a NanoDrop 2000 spectrophotometer (Thermo Fisher, USA). Samples were deemed qualified for subsequent analysis if the A260/A280 ratio was between 1.8 and 2.0. Genomic DNA contamination was eliminated during reverse-transcription reactions. For miRNA analysis, 1 µg of cDNA was synthesized using the miScript II RT kit (Qiagen, Germany) according to the manufacturer’s protocol. For lncRNA analysis, cDNA was synthesized using the RT^2^ PreAMP cDNA synthesis kit (Qiagen, Germany). GAPDH and U6 were used as internal reference genes, and the relative expression of MEG3 and miR-144-3p was determined using RT-qPCR. The relative expression of MEG3 and miR-144-3p were calculated using the 2^-ΔΔCt^ method. Three technical replicates were set for each sample, and the average value was taken as the final result. No-template controls (NTCs) were included in every qPCR run. The primers used were as follows: MEG3 forward, 5′-CTGCCCATCTACACCTCACG-3′; reverse, 5′-CTCTCCGCCGTCTGCGCTAGGGGCT-3′. miR-144-3p forward, 5′-ACACTCCAGCTGGGTACAGTAT-3′; reverse, 5′-CTCAACTGGTGTCGTGGA-3′. GAPDH forward, 5′-GAAGGTGAAGGTCGGAGTC-3′; reverse, 5′-GAAGATGGTGATGGGATTTC-3′. U6 forward, 5′-CTCGCTTCGGCAGCACA-3′; reverse, 5′-AACGCTTCACGAATTTGCGT-3′.

### Luciferase reporter gene

The target binding sites of MEG3 and miR-144-3p were identified through the starbase (http://starbase.sysu.edu.cn/) prediction. Wild-type fragments of MEG3 (MEG3-WT) and mutant fragments (MEG3-MUT) containing the binding sites were synthesized. According to the instructions of Lipofectamine 3000 (Invitrogen, USA), the recombinant vector was co-transfected with miR-144-3p mimic or negative control mimics into 293T cells (Cell Bank of Chinese Academy of Sciences) by the manufacturer. The luciferase activity was detected on a microplate reader (Thermo Fisher, USA). Three replicates were set for each experimental group, and the experiment was repeated three times. The average value was taken as the final result.

### Cell lines and culture conditions

RGCs were acquired from Zhongjiao Xinzhou Biotechnology Co., Ltd. (Shanghai, China (Shanghai, China). RGCs were cultured in DMEM/F12 medium (Gibco, California, USA), with 10% fetal bovine serum (FBS, Gibco) added. The cells were cultured in a humidified, incubator-like device at 37 °C and 5% CO_2_. The oxidative damage model was established by adding 1200 μg/mL of NaIO_3_ to the culture medium and allowing the cells to act for 24 hours (28, 29).

### Cell transfection

si-MEG3, si-NC, miR-144-3p mimic and miR-144-3p inhibitor were all purchased from Shanghai Sangon Biotech Co., Ltd. Cells were seeded in 6-well plates at a density of 2×10^7^ cells per well. Lipofectamine 3000 reagent (Invitrogen, USA) was used for transfection in accordance with the manufacturer’s instructions. Following 48 hours of transfection, the cells were gathered for further research.

### Cell viability assay

RGCs were seeded onto 96-well plates at a density of 1×10^4^ cells per well. 10 µL of Cell Counting Kit-8 (CCK-8) reagent (Sigma, USA) was added to each well, and the plates were incubated in the dark for 2 hours. The absorbance at 450 nm was quantitatively measured using a microplate reader (Thermo Fisher, USA). Each experiment was independently repeated at least three times.

### ELISA

According to the instructions of the ELISA kit (Beyotime, Shanghai), the levels of CAT, MDA and inflammatory factors (IL-6, TNF-α and IL-1β) in the AH of all the research subjects were detected. 100 μL of standard substances and samples were added to the standard substance wells and sample wells of the microplate, and then incubated at 37 °C in a constant temperature incubator for 2 hours. After washing, 100 μL of enzyme conjugate was added. Incubation was then conducted at 37 °C for 1 hour. The chromogenic agent was added, and the reaction was incubated in the dark at 37 °C for 15 minutes. After the color reaction was completed, the stop solution was added to terminate the reaction. The absorbance of each well was measured using an enzyme analyzer (Thermo Fisher, USA) at a wavelength of 450 nm.

### Screening of potential target genes of miR-144-3p

We predicted the target mRNAs of miR-144-3p using the Targetscan (https://www.targetscan.org/vert_72/), miRDB (https://mirdb.org/) and ENCORI (https://rnasysu.com/encori/). An online tool was applied to generate a Venn diagram and obtain the intersecting target genes among the three datasets. The intersecting genes were imported into the STRING database (https://cn.string-db.org/) to construct the protein-protein interaction (PPI) network. The cytohubba plugin of Cytoscape software was utilized to perform topological analysis on the PPI network, and the top 10 core target genes were screened according to the topological analysis results.

### Data analysis

Cell experiments were performed with at least three independent biological replicates. For clinical samples, each subject served as one biological replicate. All data are presented as mean ± SD. Data processing and analysis were conducted using SPSS 26.0 statistical software (IBM-SPSS, USA), and statistical graphs were drawn using GraphPad Prism 8.0 software (GraphPad Software, USA). Two-group comparisons were performed using two-tailed Student’s t-test (for normally distributed data with equal variances) or Mann-Whitney U test (for non-normally distributed data or data with unequal variances). Comparisons among three or more groups were carried out by one-way ANOVA, followed by Tukey’s multiple comparisons test for post-hoc pairwise comparisons. Categorical variables were compared using the chi-square test. Pearson correlation analysis was used to analyze the correlation between MEG3 and miR-144-3p expression. Spearman correlation analysis was used to analyze the correlations between MEG3, miR-144-3p levels and key disease indicators and inflammatory factors. Multiple testing was corrected by the Benjamini-Hochberg false-discovery rate (FDR) procedure. The diagnostic value of MEG3 and miR-144-3p in predicting early POAG was analyzed using the receiver operating characteristic (ROC) curve. The area under the curve (AUC), sensitivity, and specificity were calculated. A difference was considered statistically significant if *P* < 0.05.

## Result

### Comparison of baseline data characteristics

Baseline data comparison showed that there were no statistically significant differences in gender and age between the two groups (all *P*>0.05), while there were statistically significant differences in baseline IOP, MD and RNFL between the POAG patient group and the HC group (all *P* < 0.001). The specific baseline data are shown in **Table 1**. Furthermore, within the POAG cohort, subgroup analysis comparing early and mid-late POAG demonstrated comparable age, gender, baseline IOP and CCT between the two subgroups (all P>0.05). Mid-late POAG patients had significantly worse visual field indices, thinner RNFL, longer disease duration, and more IOP-lowering medications relative to early POAG (all P<0.001). Baseline features for POAG subgroups are summarized in **Supplementary Table 1**.

**Table 1:** Patients’ characteristics and results.

Variable	HC (n=52)	POAG (n=118)	*P* value
Age	69.37±15.80	68.50±13.54	0.716
Gender (male)	20	56	0.277
IOP (mmHg)	15.09±1.79	24.71±4.01	<0.001
MD (dB)	-0.46±0.16	-5.79±1.51	<0.001
RNFL mean (µm)	108.88±9.73	75.51±9.45	<0.001

IOP, intraocular pressure; MD, mean deviation; RNFL, retinal nerve fiber layer. Data are presented as mean ± SD.

### Verification of the targeting relationship between MEG3 and miR-144-3p

The interaction between MEG3 and miR-144-3p was predicted to be targeted through StarBase (**Figure 1A**). The dual luciferase reporter gene assay confirmed that the miR-144-3p mimic would inhibit the luciferase activity, while the miR-144-3p inhibitor would promote the luciferase activity (**Figure 1B**, all *P* < 0.001).

**Figure 1:**
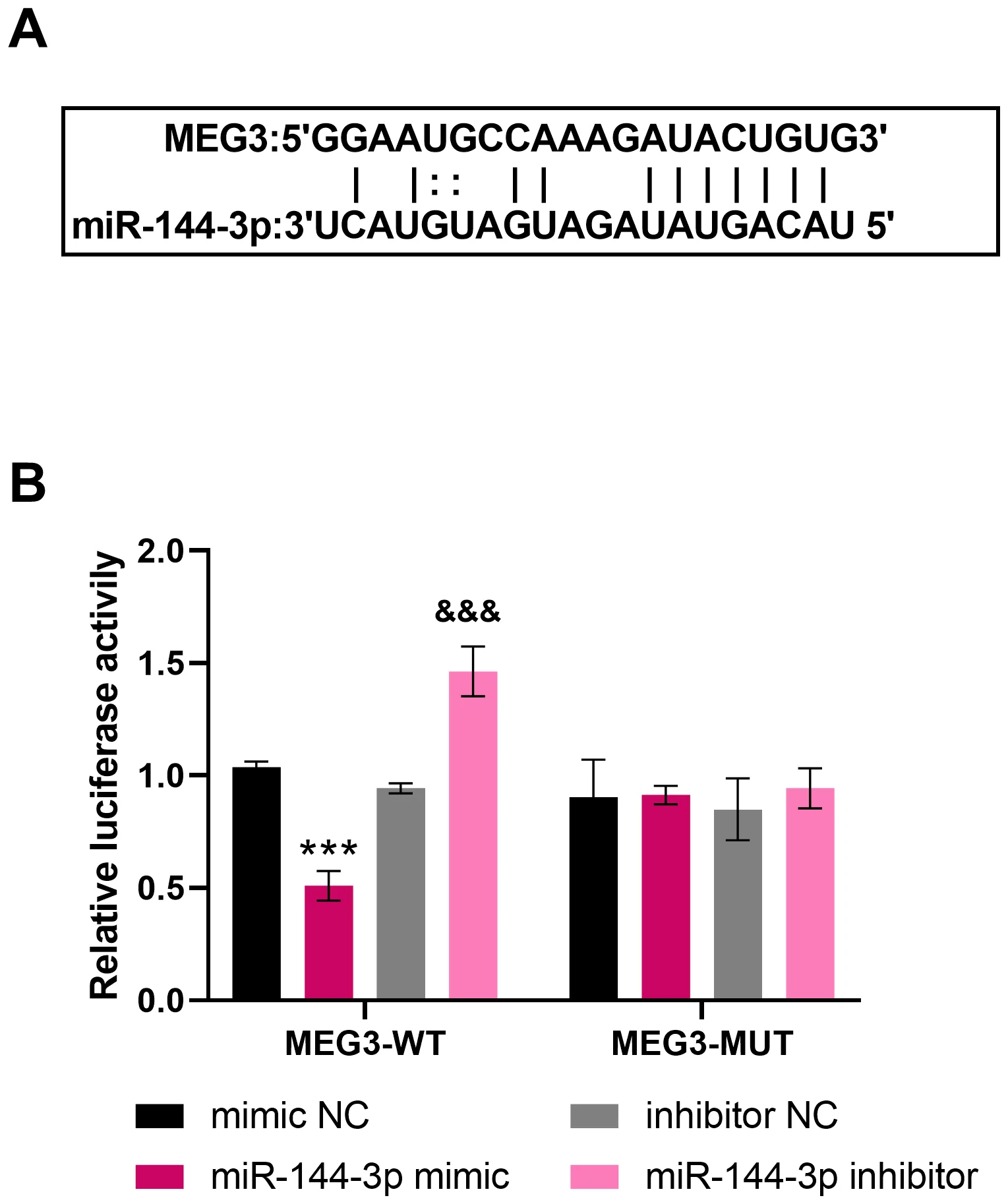
MEG3 functions as a molecular sponge for miR-144-3p. **(A)** The binding sites between MEG3 and miR-144-3p were predicted. **(B)** The luciferase assay confirmed the binding between MEG3 and miR-144-3p. Data are presented as mean ± SD, n = 3 independent experiments. Two-tailed Student’s t-test. ***P < 0.001.

### Analysis of the expression characteristics of MEG3 and miR-144-3p

The RT-qPCR results showed that in early POAG patients, the expression of MEG3 in AH was significantly higher than that in tears and serum (**Figure 2A**, *P* < 0.001). Correlation analysis confirmed that the expression of MEG3 was significantly positively correlated among the three samples (**Figures 2B–D**, adjusted *P* < 0.001). The expression of miR-144-3p showed a gradient distribution from the AH to the tears and then to the serum (**Figure 2E**, *P* < 0.001). Correlation analysis revealed that the expression of miR-144-3p was significantly positively correlated among the three sample types (**Figures 2F–H**, adjusted *P* < 0.001). We then conducted a correlation analysis on the expression of MEG3 and miR-144-3p in the AH, and found that the expressions of MEG3 and miR-144-3p were significantly negatively correlated (**Figure 2I**, *r* = -0.713, adjusted *P* < 0.001).

**Figure 2:**
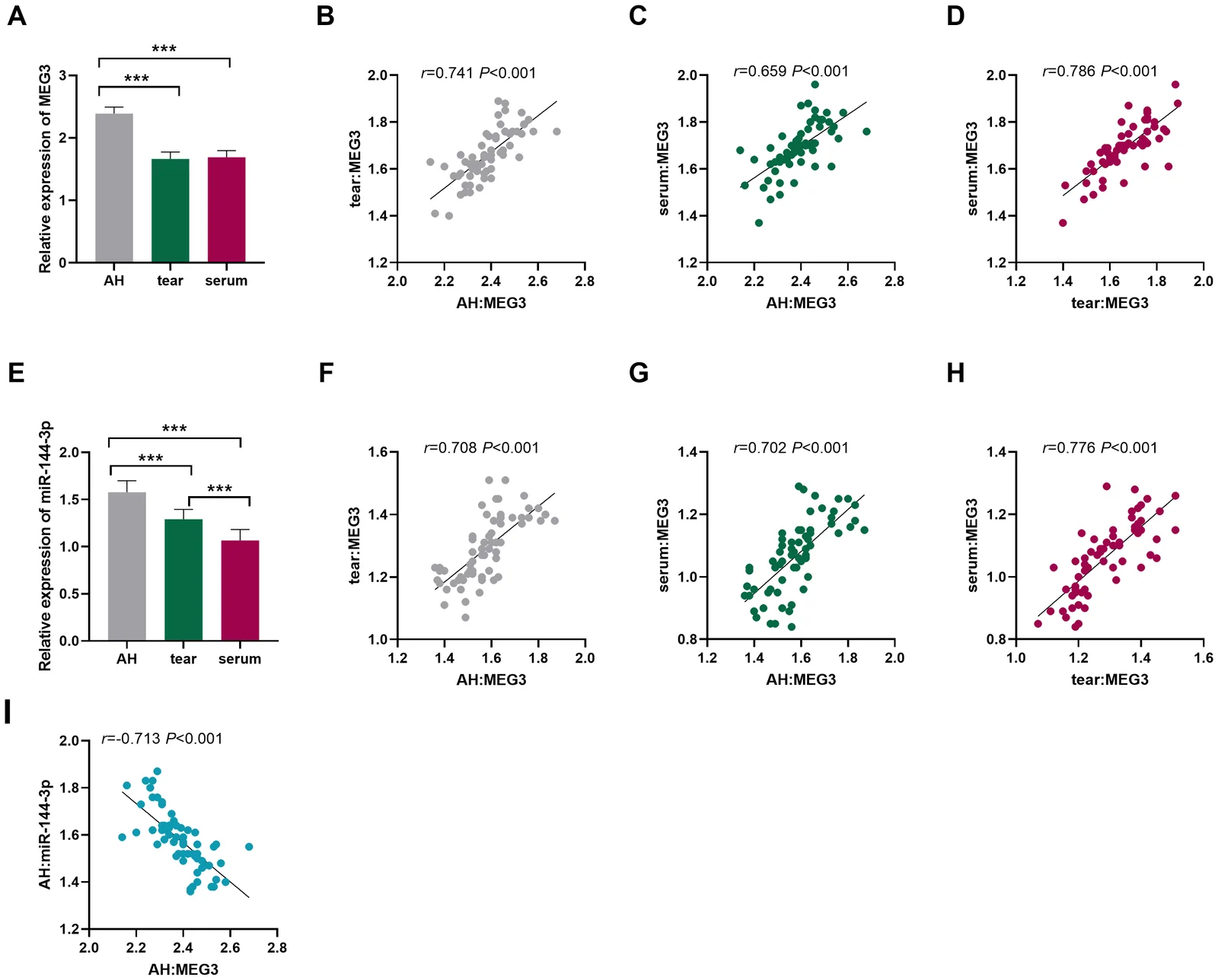
Expression and correlation of MEG3 and miR-144-3p in AH, tears and serum of early POAG patients (n = 63). **(A)** Comparison of the relative expression of MEG3 in AH, tears and serum of early POAG patients. **(B)** Correlation analysis of MEG3 in AH and tears. **(C)** Correlation analysis of MEG3 in AH and serum. **(D)** Correlation analysis of MEG3 in tears and serum. **(E)** Comparison of the relative expression of miR-144-3p in AH, tears and serum of early POAG patients. **(F)** Correlation analysis of miR-144-3p in AH and tears. **(G)** Correlation analysis of miR-144-3p in AH and serum. **(H)** Correlation analysis of miR-144-3p in tears and serum. For panel A and E: Data are presented as mean ± SD, n = 63 POAG patients. One-way ANOVA followed by Tukey’s multiple comparisons test. For panels **(B–D, F–H)** Spearman correlation analysis. ***P < 0.001.

### Diagnostic efficacy of MEG3 and miR-144-3p in early POAG

The results showed that compared with the HC, the expression of MEG3 in the tears and serum of early POAG patients were significantly increased (**Figures 3A, B**, *P* < 0.001), while the expression of miR-144-3p was significantly decreased (**Figures 3C, D**, *P* < 0.001). ROC curve analysis revealed that the AUC of MEG3 in tears was 0.894, with a cutoff value of 1.445, a sensitivity of 88.89%, and a specificity of 84.62% (**Figure 3E**). The AUC of MEG3 in serum was 0.899, with a cutoff value of 1.265, a sensitivity of 90.48%, and a specificity of 76.92% (**Figure 3F**). The AUC of miR-144-3p in tears was 0.873, with a cutoff value of 1.425, a sensitivity of 85.71%, and a specificity of 78.85% (**Figure 3G**). The AUC of miR-144-3p in serum was 0.877, with a cutoff value of 1.285, a sensitivity of 90.48%, and a specificity of 73.08% (**Figure 3H**). Further analysis of the combined diagnostic efficacy of the two methods revealed that the AUC for the combined detection of MEG3 in tears and miR-144-3p was 0.914, and the AUC for the combined detection of serum was 0.910 (**Figures 3I, J**).

**Figure 3:**
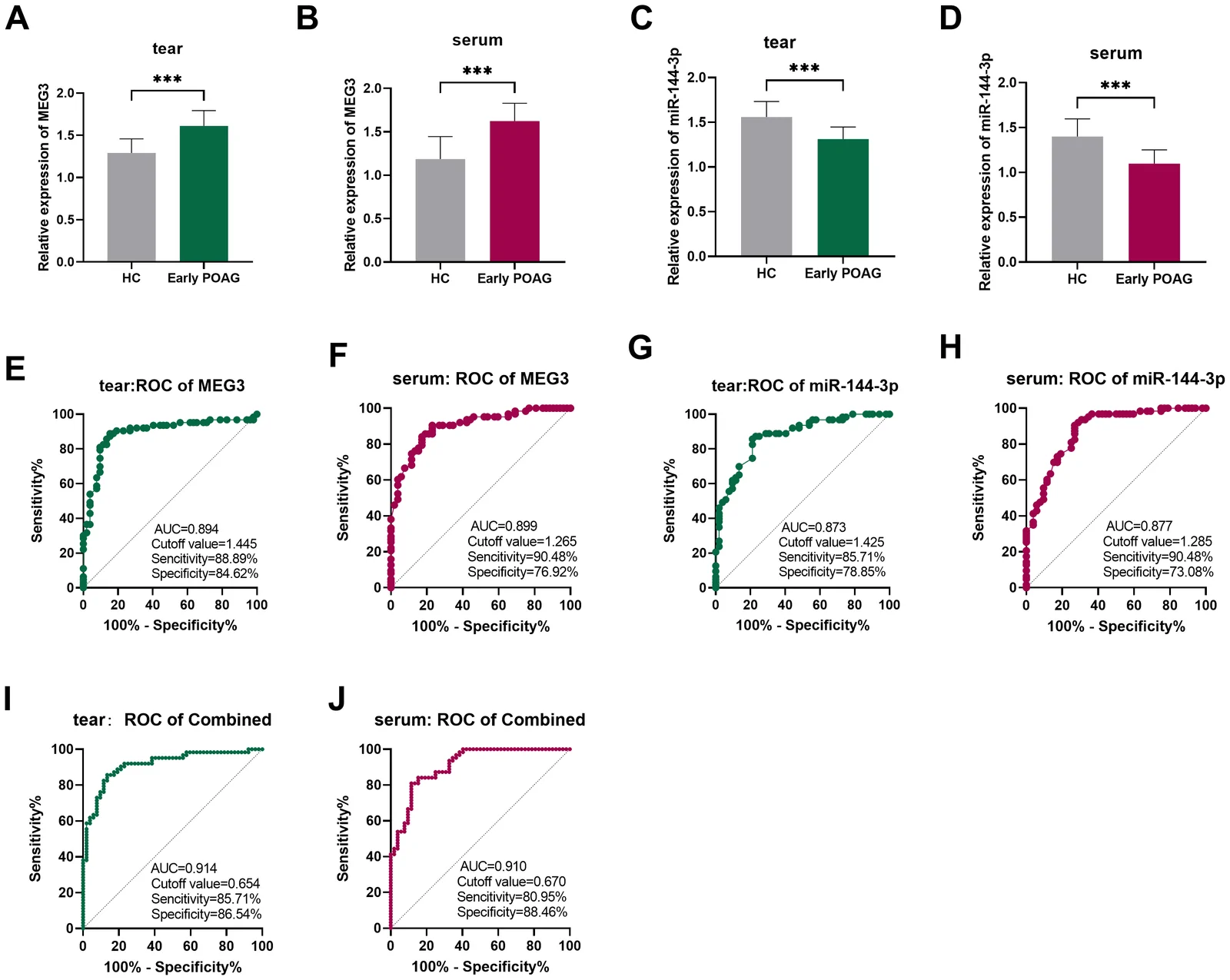
Expression and diagnostic value of MEG3 and miR-144-3p in tears and serum samples from early POAG patients. **(A–D)** Relative expression of MEG3 and miR-144-3p in tear **(A, C)** and serum **(B, D)** samples from HC and early POAG patients. **(E–H)** ROC curves evaluating the diagnostic performance of MEG3 **(E, F)** and miR-144-3p **(G, H)** in tears and serum samples, respectively. **(I)** ROC curve of the combined expression of MEG3 and miR-144-3p in tears. **J**. ROC curve of the combined expression of MEG3 and miR-144-3p in serum. Data are presented as mean ± SEM, n = 52 for HC group, n = 63 for POAG group. Statistical comparisons between the two groups were performed using Student’s t-test. ***P < 0.001, compared with HC group.

### Analysis of MEG3 and miR-144-3p in relation to disease severity

We also investigated the changes in the expression of MEG3 and miR-144-3p in AH, tears, and serum among patients with early and mid-late POAG. The results showed that MEG3 expression in AH, tears, and serum of patients with mid-late POAG was significantly higher than that in early POAG patients (**Figures 4A–C**, *P* < 0.001). Conversely, miR-144-3p expression in the AH, tears, and serum of patients with mid-late POAG was significantly lower than that in early POAG patients (**Figures 4D–F**, *P* < 0.001).

**Figure 4:**
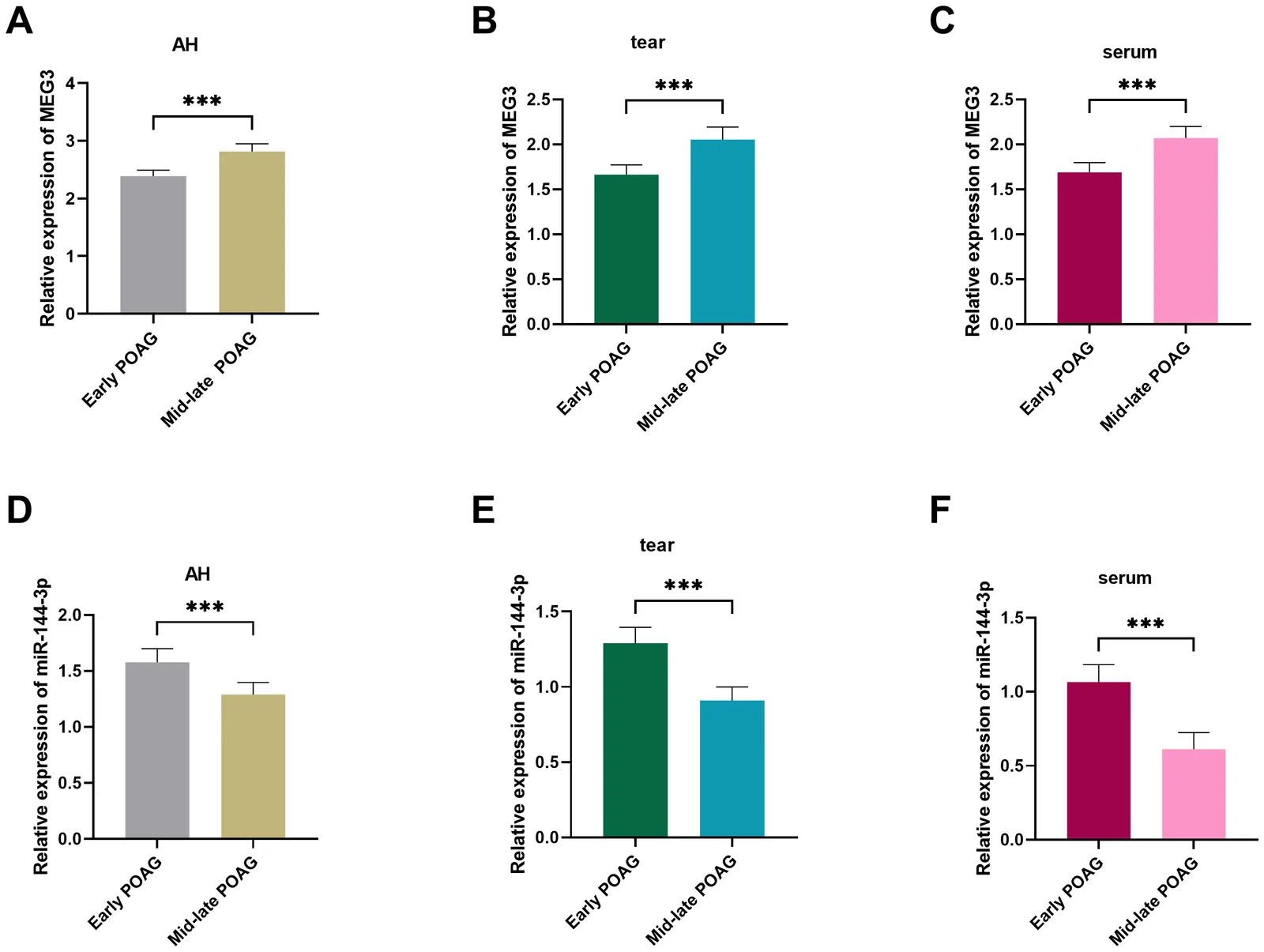
Expression of MEG3 and miR-144-3p in AH, tears, and serum samples from early and mid-late POAG patients. **(A–C)** Relative expression of MEG3 in AH **(A)**, tears **(B)**, and serum **(C)** samples from early and mid-late POAG patients. **(D–F)** Relative expression of miR-144-3p in AH **(D)**, tears **(E)**, and serum **(F)** samples from early and mid-late POAG patients. Data are presented as mean ± SEM, n = 63 for early POAG group, n = 55 for mid-late POAG group. Statistical comparisons between the two groups were performed using Student’s t-test. ***P < 0.001, compared with early POAG group.

### Correlation analysis of MEG3 and miR-144-3p with clinical indicators and inflammatory factors

As shown in **Figure 5**. In the AH of patients with early POAG, MEG3 was significantly positively correlated with IOP (*r* = 0.777, adjusted *P* < 0.001), MD (*r* = 0.688, adjusted *P* < 0.001), IL-6 (*r* = 0.680, adjusted *P* < 0.001), IL-1β (*r* = 0.728, adjusted *P* < 0.001), and TNF-α (*r* = 0.712, adjusted *P* < 0.001), while it was significantly negatively correlated with RNFL (*r* = -0.716, adjusted *P* < 0.001). miR-144-3p was significantly negatively correlated with IOP (*r* = -0.811, adjusted *P* < 0.001), MD (*r* = -0.670, adjusted *P* < 0.001), IL-6 (*r* = -0.835, adjusted *P* < 0.001), IL-1β (*r* = -0.736, adjusted *P* < 0.001), and TNF-α (*r* = -0.752, adjusted *P* < 0.001), and miR-144-3p was significantly positively correlated with RNFL (*r* = 0.812, adjusted *P* < 0.001).

**Figure 5:**
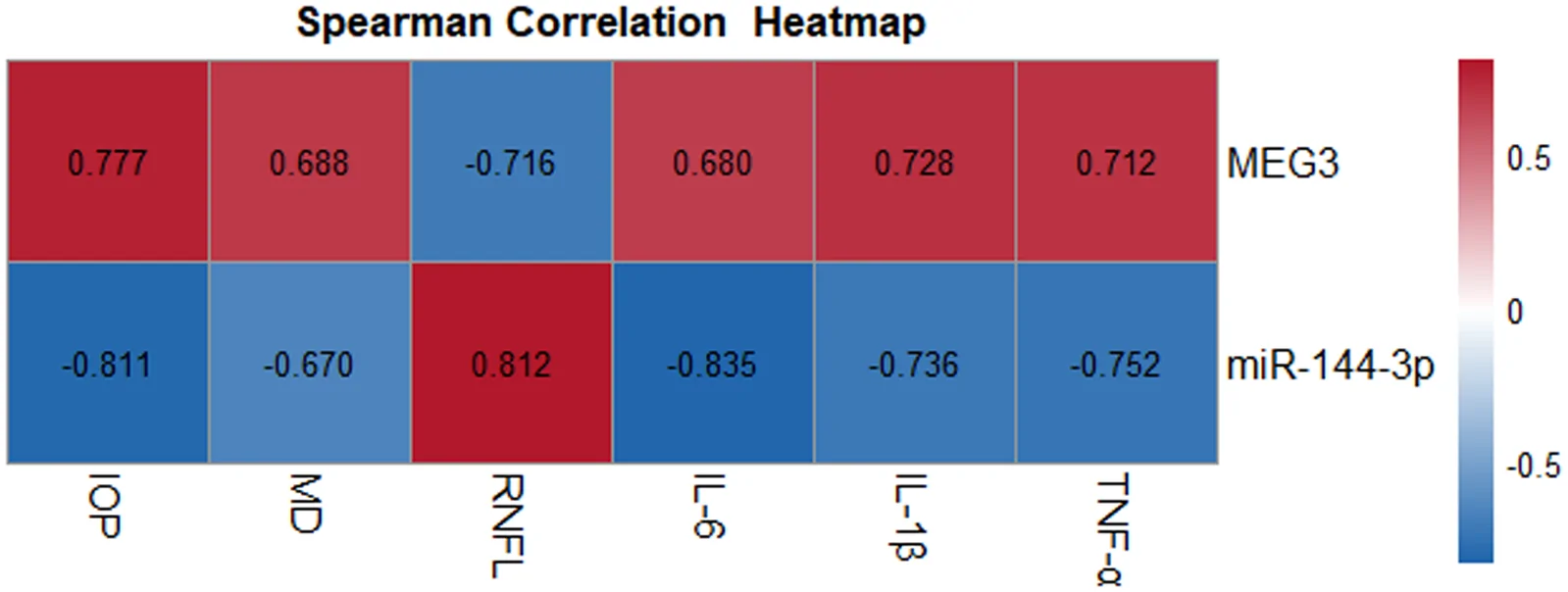
Spearman correlation heatmap of MEG3, miR-144-3p, and clinical, inflammatory indicators in POAG patients. The heatmap shows Spearman correlation coefficients between MEG3/miR-144-3p expression and glaucoma-related clinical indicators (IOP, MD, RNFL) as well as inflammatory cytokines (IL-6, IL-1β, TNF-α). Red indicates a positive correlation, and blue indicates a negative correlation, with the color intensity reflecting the strength of the correlation. The numerical values in each cell represent the correlation coefficient *r*. Spearman correlation analysis was used to evaluate the correlations among the various indicators. Multiple tests were corrected using the Benjamini-Hochberg false discovery rate (FDR) method. A two-tailed adjusted *P* < 0.05 was considered to indicate statistical significance.

### The expression and function of MEG3 in the oxidative stress cell model

In the oxidative stress model induced by NaIO_3_, we investigated the regulatory effects of MEG3 on cell damage, inflammatory response, and oxidative stress indicators. The results showed that NaIO_3_ treatment significantly upregulated the expression of MEG3 in the cells (**Figure 6A**, *P* < 0.001), while the cell survival rate significantly decreased (**Figure 6B**, *P* < 0.001), the secretion levels of inflammatory factors IL-6, IL-1β, and TNF-α significantly increased (**Figure 6C**, *P* < 0.001), and the levels of oxidative stress indicators CAT and MDA also significantly rose (**Figure 6D**, *P* < 0.001). After transfection with si-MEG3, the expression of MEG3 was significantly downregulated (**Figure 6A**, *P* < 0.001), the cell survival rate significantly increased (**Figure 6B**, *P* < 0.001), the release of inflammatory factors was significantly inhibited (**Figure 6C**, *P* < 0.001), and the levels of CAT and MDA also significantly decreased (**Figure 6D**, *P* < 0.001).

**Figure 6:**
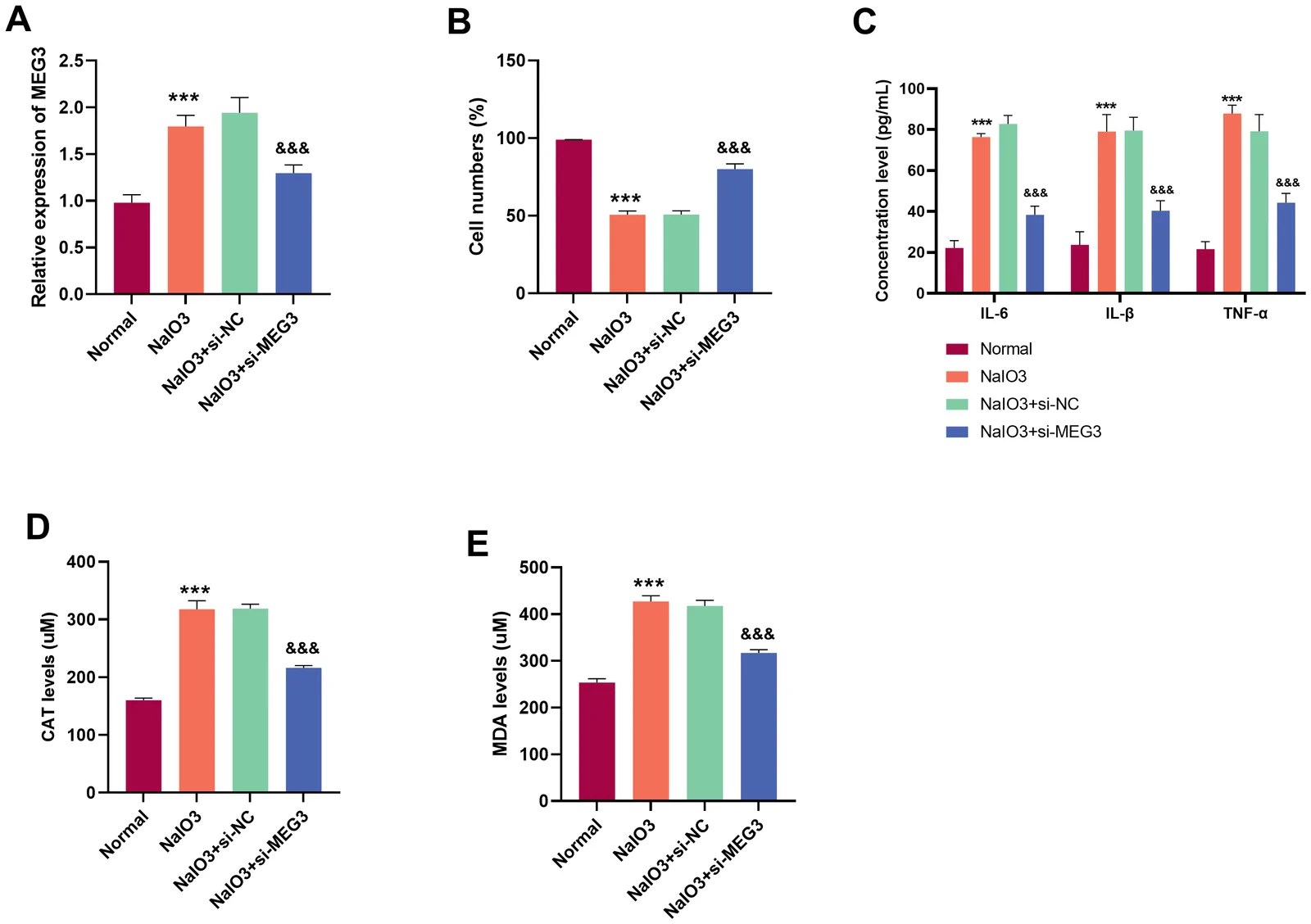
Regulation of MEG3 on cell function and oxidative stress. **(A)** Relative expression of MEG3 in each group of cells. **(B)** Cell viability detected after different treatments. **(C)** Concentrations of inflammatory factors in cell supernatants. **(D, E)** CAT activity and MDA levels in cells, reflecting oxidative stress status. Data are presented as mean ± SEM, n = 3 independent experiments. Comparisons among multiple groups were performed using one-way ANOVA followed by Tukey’s multiple comparisons test. ***P < 0.0001, compared with the Normal group; &&& *P* < 0.001, compared with the NaIO_3_+si-NC group.

### miR-144-3p mediates the regulation of MEG3 on cell viability and oxidative stress

We investigated the mutual regulatory interaction between MEG3 and miR-144-3p. As shown in **Figure 7A**, the NaIO_3_ modeling treatment significantly downregulated the expression of miR-144-3p in the cells (*P* < 0.001). Transfection with si-MEG3 significantly promoted the expression of miR-144-3p (*P* < 0.001), while transfection with miR-144-3p mimic also significantly upregulated the expression of miR-144-3p (*P* < 0.001). However, transfection with miR-144-3p inhibitor could inhibit the promoting effect of si-MEG3 on the expression of miR-144-3p (*P* < 0.001). Overexpression of miR-144-3p could significantly reverse the damaging effect of NaIO_3_ model treatment on the cells. Compared with the NaIO_3_ group, overexpression of miR-144-3p significantly increased cell survival rate (**Figure 7C**, *P* < 0.001), inhibited the secretion of IL-6, IL-1β, and TNF-α (**Figure 7D**, *P* < 0.001), and downregulated the levels of oxidative stress indicators CAT and MDA (**Figures 7E, F**, *P* < 0.001). Further studies showed that miR-144-3p inhibitor could significantly reverse the effect of si-MEG3 on cell function, manifested as decreased cell survival rate, increased secretion of inflammatory factors, and increased oxidative stress levels (**Figures 7C–F**, *P* < 0.001).

**Figure 7:**
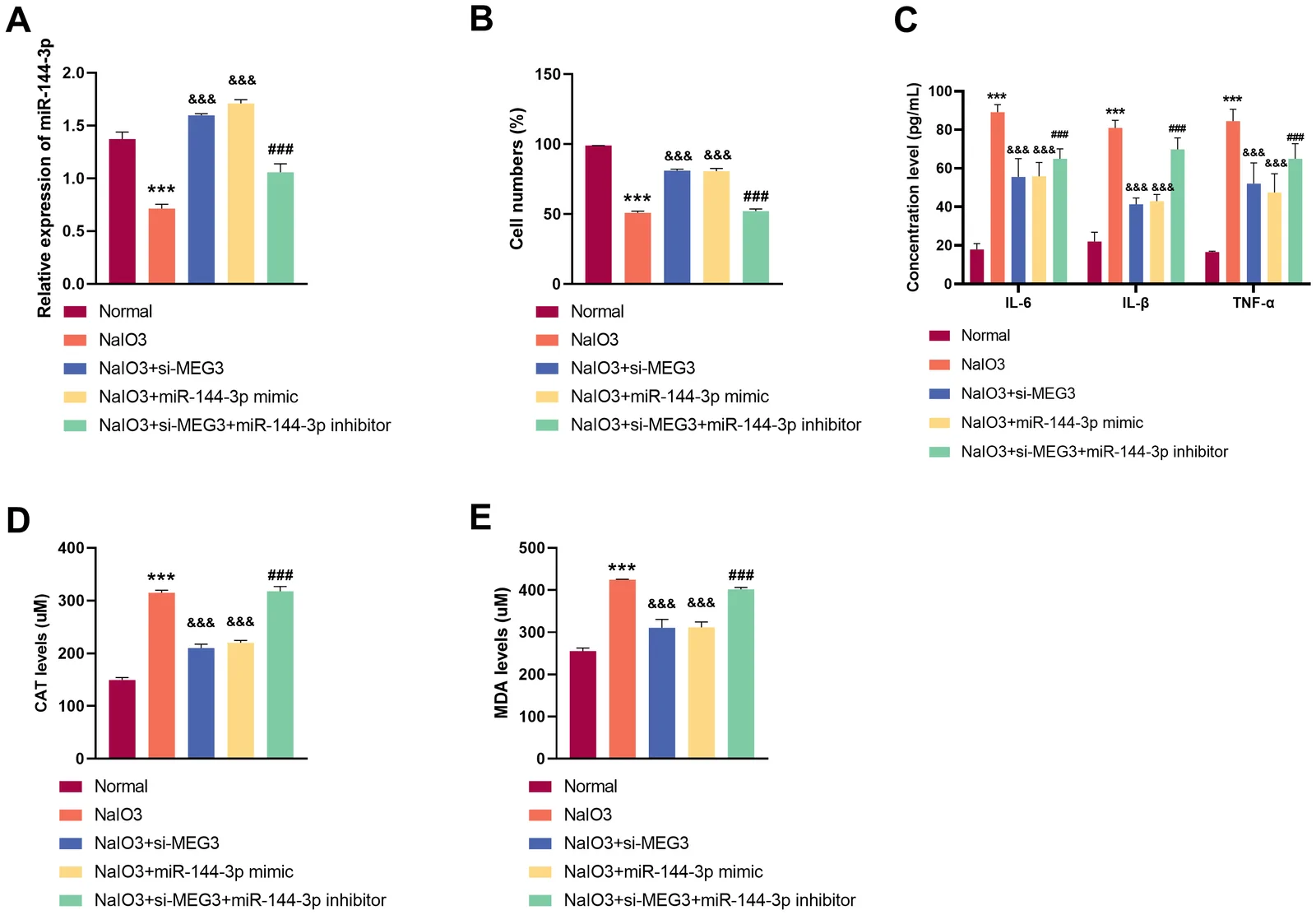
miR-144-3p mediates the regulation of MEG3 on cell viability and oxidative stress. **(A)** Relative expression of miR-144-3p in each treatment group. **(B)** Cell viability detected after different treatments. **(C)** Concentrations of inflammatory factors in cell supernatants. **(D, E)** CAT activity and MDA levels in cells, reflecting oxidative stress status. Data are presented as mean ± SEM, n = 3 independent experiments. Comparisons among multiple groups were performed using one-way ANOVA followed by Tukey’s multiple comparisons test. ***P < 0.001, compared with the Normal group; &&&*P* < 0.001, compared with the NaIO_3_ group; ###*P* < 0.001, compared with the NaIO_3_+si-MEG3 group.

### Prediction of potential target genes of miR-144-3p

We used the public databases Targetscan, miRDB and ENCORI to predict the potential target genes of miR-144-3p. As shown in the Venn diagram, the three databases jointly identified 487 overlapping target genes (**Figure 8A**). PPI network reveals complex regulatory connections among these 487 potential target genes (**Figure 8B**). Further analysis of the network using the CytoHubba plugin of Cytoscape software yielded the top 10 Hub genes, including ARID1A, RUNX1, EZH2, DNMT3A, IDH2, JAK2, TET2, PTEN, PHF6, and CEBPA (**Figure 8C**).

**Figure 8:**
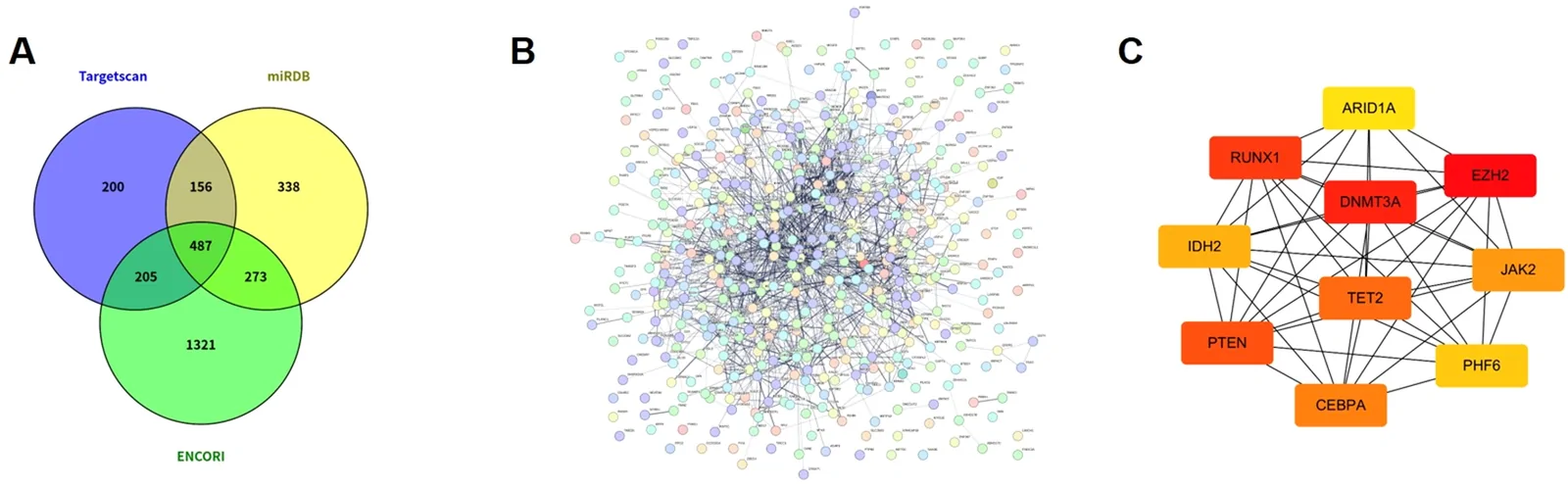
Prediction of potential target genes of miR-144-3p. **(A)** Venn diagram showing the overlapping target genes of miR-144-3p predicted by three online databases, TargetScan, miRDB, and ENCORI. **(B)** PPI network of the intersected target genes of miR-144-3p. **(C)** Hub genes screened from the PPI network.

## Discussion

POAG has a complex pathogenesis (30). Oxidative stress and inflammatory responses have been confirmed to be one of the key pathological processes in POAG (31). Currently, there are no specific early diagnostic markers or targeted therapeutic targets in clinical practice. Exploring the key molecular regulatory network during the progression of POAG is extremely important for improving disease diagnosis and treatment (32). This study suggests that in POAG, MEG3 can target and regulate miR-144-3p, and it is expected to become a potential diagnostic marker for this disease.

This study confirmed that MEG3 has a direct target binding relationship with miR-144-3p, and the two have a significant negative correlation in AH (*r* = -0.713). This finding supports the classic regulatory model of lncRNA as a ceRNA sponge that adsorbs miRNAs, laying the groundwork for further investigation of their roles in POAG (33). Previous research has shown that MEG3 can participate in disease pathology by targeting miRNA binding, and its regulatory effects are primarily associated with pathological mechanisms such as inflammatory responses and oxidative stress. For instance, MEG3 can activate the NF-κB pathway by adsorbing miR-21, thereby promoting oxidative stress and cell apoptosis induced by inflammatory factors (34). MEG3 can also specifically bind to miR-34a, inhibiting the secretion of inflammatory factors such as IL-1β, IL-6 and TNF-α in RGCs (35). This study further revealed that MEG3 showed a significant positive correlation in the AH, tears and serum of POAG patients. Compared with the HC, the expression of MEG3 in the tears and serum of patients was significantly upregulated. The ROC analysis results indicated that MEG3 has the potential to be a potential biomarker for the diagnosis of early POAG. Abnormal expression of miR-144-3p, a key regulator of ocular inflammation and oxidative stress responses, has been linked to a number of ocular diseases. Wei et al. discovered that miR-144-3p was abnormally upregulated in the diabetic corneal lesion model, and that inhibiting its expression could promote corneal epithelial repair and nerve regeneration, implying that it plays an important regulatory role in the pathogenesis of ocular diseases (36). Cao et al. found that miR-144-3p was reduced in glaucoma tissues and human Tenon’s capsule fibroblasts treated with TGF-β1 (27). This indicates that the expression changes of miR-144-3p may be closely related to the pathological mechanism of glaucoma. Our analysis of this study on POAG patients revealed that miR-144-3p was distributed in a gradient pattern in the patients’ AH, tears, and serum, which was highly consistent with the intraocular microenvironmental damage characteristics. Further ROC curve analysis confirmed that miR-144-3p in tears and serum had high early POAG diagnostic efficacy. For serum samples, the cutoff value of MEG3 was 1.265, the cutoff value of miR-144-3p was 1.285, and the combined detection achieved an AUC of 0.910 at a cutoff of 0.670, which outperformed individual biomarkers. In clinical practice, these thresholds can serve as reference values for early diagnosis in hospitals. The serum test results can be compared with these critical values to assist clinicians in identifying patients suspected of having POAG. However, before these thresholds are formally applied in clinical settings, they still need to undergo external validation in larger independent cohorts.

Oxidative stress and inflammatory response are the core pathological mechanisms of POAG. The oxidative damage of trabecular meshwork cells and RGCs is a key link in the disease progression (37, 38). This study used an oxidative stress cell model established by NaIO_3_ on RGCs to confirm that MEG3 expression was significantly upregulated under oxidative stress conditions. Silencing MEG3 could significantly alleviate cell viability inhibition, reduce the release of inflammatory factors (IL-6, TNF-α, IL-1β) and the levels of oxidative stress indicators (CAT, MDA). This is consistent with the previous research results. Previous research discovered that MEG3 activates the Sirt1 pathway by targeting miR-204 to alleviate oxidative stress and inflammation in Muller cells in diabetic retinopathy (39). NaIO_3_ treatment significantly upregulated MEG3 and inhibited the expression of miR-144-3p. Overexpression of miR-144-3p could reverse the oxidative stress and inflammatory effects of MEG3, while inhibiting miR-144-3p could counteract the effect of silencing MEG3. This regulatory pattern is consistent with the anti-inflammatory and antioxidant function of miR-144-3p. Previous studies have shown that miR-144 can target and regulate inflammatory-related pathways and play an anti-inflammatory role in various inflammatory diseases, and its abnormal expression is closely related to the release of inflammatory factors (40, 41).

This study still has certain limitations. Firstly, this study is a single-center cohort study with a relatively small sample size. Although ROC analysis indicated that MEG3/miR-144-3p has promising diagnostic performance, independent external validation and larger prospective cohort studies are required before MEG3/miR-144-3p can be considered clinically applicable biomarkers. Secondly, this study only conducted bioinformatics prediction of the downstream target genes of miR-144-3p, without further exploring the complete molecular pathway of this regulatory axis. Finally, mechanistic investigations in this study were based on the NaIO_3_-induced oxidative-stress model in retinal pigment epithelial cells. This model cannot fully recapitulate the pathological features of POAG, and we lack animal models that mimic intraocular pressure elevation, choroidal reticular structure dysfunction, and optic nerve degeneration. We will conduct animal experiments to confirm the regulatory axis’s *in vivo* effects, investigate the therapeutic potential of miR-144-3p mimics or MEG3 inhibitors, and develop new strategies for the targeted treatment of POAG.

## Conclusion

Our findings indicate that MEG3 and miR-144-3p may serve as potential non-invasive biomarkers for early POAG diagnosis. The MEG3/miR-144-3p axis may be involved in the pathogenesis of glaucoma by regulating RGCs survival, inflammatory responses and oxidative stress, providing new insights into the molecular mechanisms of glaucoma and offering potential directions for early diagnosis and targeted therapy.

## Data Availability

The datasets presented in this study can be found in online repositories. The names of the repository/repositories and accession number(s) can be found in the article/supplementary material.
