# Supplementary material for: Clinical value evaluation of MEG3/miR-144-3p as a biomarker for the early diagnosis of glaucoma
Source: Front Immunol. 2026 Oct 20;17:1894482. doi: 10.3389/fimmu.2026.1894482 (PMC13727485; doi:10.3389/fimmu.2026.1894482)
Supplement: Supplementary file 1 [file SupplementaryFile1.docx]

Supplementary Table 1. Baseline clinical characteristics of patients with early and mid‑late POAG

| Variable | Early POAG (n=63) | Mid-late POAG (n=55) | *P* value |
| --- | --- | --- | --- |
| Age | 68.03±13.17 | 69.04±14.06 | 0.690 |
| Gender (male) | 29 | 27 | 0.740 |
| Baseline IOP (mmHg) | 24.78±4.17 | 24.52±4.01 | 0.737 |
| MD (dB) | -4.64±1.03 | -7.09±1.02 | <0.001 |
| RNFL mean (µm) | 81.51±6.82 | 68.64±7.07 | <0.001 |
| Disease duration (years) | 4.02±1.70 | 6.08±2.03 | <0.001 |
| Number of IOP-lowering medications | 1.66±0.79 | 2.38±1.03 | <0.001 |
| CCT (µm) | 521.35±45.38 | 524.31±45.89 | 0.726 |
| Visual field sensitivity | 26.1±4.87 | 19.18±5.32 | <0.001 |

Note: POAG, primary open‑angle glaucoma; IOP, intraocular pressure; MD, mean deviation; RNFL, retinal nerve fiber layer; CCT, central corneal thickness. Continuous variables are presented as mean ± standard deviation. Independent‑samples t‑test was used for continuous variables; Chi‑square test for categorical variables.
